# Targeting complex IV to alter the tumor microenvironment and boost macrophage antitumor immunity

**DOI:** 10.1093/lifemeta/loaf032

**Published:** 2025-08-01

**Authors:** Thomas Pfefer, Luke A J O’Neill

**Affiliations:** School of Biochemistry and Immunology, Trinity Biomedical Sciences Institute, Trinity College Dublin, Dublin D02 R590, Ireland; School of Biochemistry and Immunology, Trinity Biomedical Sciences Institute, Trinity College Dublin, Dublin D02 R590, Ireland

## Abstract

Clark *et al*. showcase that interferons (IFNs) trigger a functional reprogramming of tumor-associated macrophages (TAMs) by downregulating NADH dehydrogenase (ubiquinone) 1 alpha subcomplex 4 (NDUFA4), a key subunit of mitochondrial complex Ⅳ. This drives a transition from protumor TAMs to antitumor IFN-associated TAMs (IFN-TAMs) through activation of the cyclic GMP-AMP synthase–stimulator of interferon genes (cGAS–STING) pathway. This mechanism can be leveraged to boost antitumor immunity and improve responses to immune checkpoint blockade.

The tumor microenvironment (TME) dynamically influences immune cells to either assist or resist cancer progression. Tumor-associated macrophages (TAMs) are particularly receptive to modulatory cues from the TME, and are often rewired to exacerbate tumor-supporting angiogenesis and inhibit key antitumor effector cells, such as cytotoxic T cells and natural killer (NK) cells [1]. Single-cell RNA sequencing (scRNA-seq) has revealed great heterogeneity among TAMs, some of which display protumor properties, while others, such as a subset referred to as interferon (IFN)-associated TAMs (IFN-TAMs), have antitumor effects [2]. The ratio of these TAM populations in the TME is predictive of disease severity and the efficacy of immune checkpoint blockade therapies [3].

What determines whether a macrophage will promote or oppose tumor development is a key question in the field of antitumor immunity. Intriguingly, these subsets often reside in spatially distinct regions of tumors, hinting that their identity may be shaped by localized environmental cues [3, 4]. A study by Clark *et al*. in *Immunity* identifies complex IV in the electron transport chain (ETC) in macrophage mitochondria as a responder to IFNs in the TME, which in turn drives IFN and chemokine production, thereby promoting antitumor immunity [5].

Complex IV is the last complex in the ETC, with oxygen acting as the terminal electron acceptor, further promoting the proton gradient necessary for ATP production. Complex IV is unique among ETC complexes, since some components are encoded by the mitochondrial genome, whereas others are encoded by nuclear genes.

The authors began with scRNA-seq of TAMs isolated from B16-F10 melanoma tumors and their splenic monocyte precursors. They discovered that a highly conserved bifunctional nuclear transcript was upregulated in IFN-TAMs, termed AA467197 in mice. This was corroborated by the analysis of a human single-cell atlas spanning multiple cancer types, with the human variant of AA467197, C15orf48, being upregulated in pro-inflammatory TAMs. Importantly, the transcript encodes a complex IV component termed NADH dehydrogenase (ubiquinone) 1 alpha subcomplex 4 (NDUFA4)-Like 3 (NDUFA4L3), which is an isoform of the well-characterized NDUFA4, and a microRNA, miR-147, which targets *Ndufa4* mRNA. This dual-purpose molecule allows for the degradation and replacement of NDUFA4 within complex IV by NDUFA4L3. NDUFA4L3 expression was shown to increase in the B16 melanoma model, correlating with the major histocompatibility complex class Ⅱ (MHC-Ⅱ) expression, which is associated with antitumor immunity.

Several genetically modified mouse models were then constructed to test the role of these components in antitumor immunity. Deletion of *Ndufa4* resulted in a significant expansion of IFN-TAMs expressing MHC-Ⅱ and a corresponding decrease in protumor TAMs, leading to reduced tumor growth across multiple tumor models, including B16 melanoma, EL-4 lymphoma, and the PDAC pancreatic cancer model. On the other hand, mice deficient in miR-147 or NDUFA4L3 exhibited elevated NDUFA4 levels, diminished IFN-TAM populations, and accelerated tumor progression.

To understand the contribution of *Ndufa4* deletion in immune cells, the researchers then transplanted bone marrow from wild-type or *Ndufa4*-deficient mice into wild-type hosts that were lethally irradiated to remove their hematopoietic cells. After implanting these mice with B16-F10 melanoma, they found that *Ndufa4*-deficient immune cells were able to slow tumor growth and had more MHC-Ⅱ + IFN-TAMs than their wild-type counterparts. Finally, to confirm that the antitumor effect was due to macrophages, the researchers generated *Ndufa4*-floxed mice crossed with LysM-Cre or S100a8-Cre drivers to conditionally delete *Ndufa4* in macrophages or neutrophils, respectively. They found reduced tumor growth and increased MHC-Ⅱ + TAM only in macrophage-specific *Ndufa4* knockout mice. Altogether, they show that macrophages are the key effectors of this TME reprogramming from NDUFA4 deficiency.

These antitumor macrophages were shown to be regulated by type Ⅰ and Ⅱ IFNs, which occur in the TME. Upon stimulation with IFN-γ, bone marrow-derived macrophages increased the expression of the bifunctional transcript, leading to reduced NDUFA4 protein levels and elevated NDUFA4L3. Using immunofluorescence microscopy, the authors investigated mitochondrial stress in these cells, examining nucleoids and the storage units of mitochondrial DNA (mtDNA), which are released from the mitochondria into the cytosol during mitochondrial stress. They found that IFN-γ-treated *Ndufa4*-deleted macrophages exhibited larger nucleoid areas and greater amounts of cytosolic mtDNA, where it was likely sensed by the pro-inflammatory cyclic GMP-AMP synthase–stimulator of interferon genes (cGAS–STING) system to boost the expression of IFN-sensitive genes (ISGs). Ablating STING or inhibiting cGAS in tumor-bearing *Ndufa4*-deficient mice restored tumor growth. The authors therefore concluded that IFN-triggered NDUFA4 remodeling destabilizes complex IV, resulting in mitochondrial stress and subsequent mtDNA release into the cytosol, which activates the cGAS–STING pathway, amplifying the type Ⅰ IFN signal and upregulating ISGs such as C-X-C motif chemokine ligand 9 (CXCL9), strengthening the IFN-TAM phenotype.

Tumors in mice lacking *Ndufa4* were shown via scRNA-seq and flow cytometry to have increased infiltration of NK cells and improved activation of CD8^+^ T cells, with improved expression of IFN-γ and cytotoxic molecules, and decreased markers of T-cell exhaustion. They tested blocking C-X-C motif chemokine receptor 3 (CXCR3), the chemokine receptor for CXCL9/10, which reversed these effects, supporting that chemokines secreted by IFN-TAMs mediate the recruitment and activation of effector lymphocytes to restrict tumor growth.

Finally, the authors explored therapeutic prospects by creating a synthetic miR-147 mimic, which was delivered intratumorally together with anti-PD-1 antibodies. The synthetic miR-147 significantly slowed tumor growth in otherwise anti-PD-1-resistant B16-F10 melanoma, boosting immune cell infiltration and CD8^+^ T-cell activation (Fig. 1).

**Figure 1:**
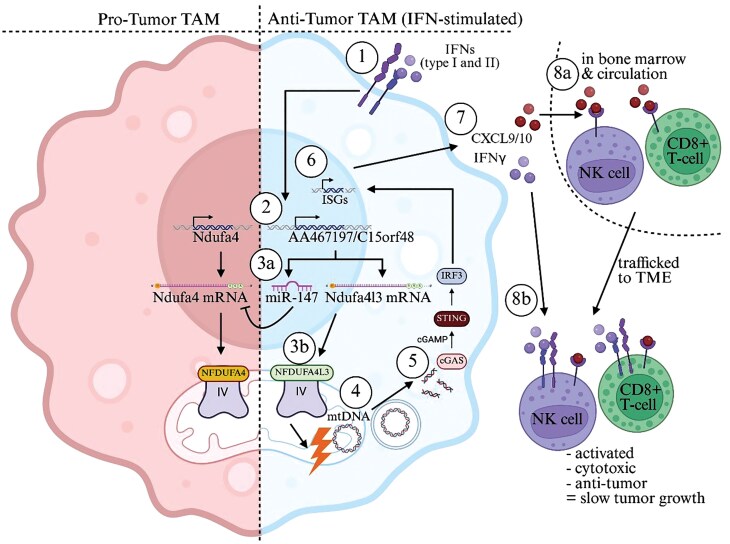
IFN-driven mitochondrial rewiring activates TAMs, impeding tumor growth. Clark *et al*. demonstrate that following type Ⅰ and Ⅱ IFN signaling (1), TAMs upregulate the bifunctional transcript AA467197 (mouse)/C15orf48 (human) (2) [5]. This transcript is made up of a microRNA, miR-147, that degrades *Ndufa4* mRNA (3a) and *Ndufa4l3*, an isoform of NDUFA4 that can then replace it as the functional subunit of Complex IV in the ETC (3b). This subunit switch induces mitochondrial stress and incidental mtDNA release (4), which is then sensed by the cGAS–STING system (5), ultimately upregulating ISGs (6). These reprogrammed TAMs then secrete CXCL9/10 and IFN-γ (7) that recruit lymphocytes from the periphery (8a) and activate them in the TME (8b), respectively. Altogether, this mechanism biases TAMs to orchestrate antitumor immunity.

IFNs are therefore driving what is called anterograde signaling from the nucleus to the mitochondria, upregulating the expression of a bifunctional transcript encoding miR-147 and NDUFA4L3; miR-147 targets the NDUFA4 subunit of complex IV, which is then substituted by NDUFA4L3, leading to ETC destabilization and mtDNA cytosolic release. This activates the cGAS–STING DNA-sensing system, leading to elevated transcription of ISGs. In the context of cancer, this culminates in a global reshaping of the TME, expanding the population of IFN-expressing TAMs, which release IFNs to promote antitumor immunity, and secrete CXCL9/10 and other chemokines to recruit and activate more immune cells to combat tumor growth. Exactly how NDUFA4 and NDUFA4L3 differentially modulate complex IV function to lead to mtDNA release will require further investigation.

By harnessing miRNA precision or using other approaches, this work could lay the foundation for establishing a new class of metabolic immunotherapies that target complex IV to treat cancer, which would be predicted to be especially effective in combination with checkpoint blockade.
